# Abstracts &c.

**Published:** 1889-08

**Authors:** 


### ABSTRACTS AND GLEANINGS.

---

**Prevention of Bedsores.**—Prof. Rosenbach regards lanolin as a normal constituent of the human skin, and able to prevent the entrance of destructive agents into the tissues. When patients are obliged to keep to their beds for a long time he prefers, after having fully disinfected the tender parts, to rub lanolin in thoroughly, after which he covers them with jute, oakum or some similar material to prevent any further pressure. During the last nine months this treatment has been applied by him in a large number of chronic affections. When the excoriations and the suspicious redness of the skin were already present, healing took place kindly under the lanolin treatment. Since adopting this treatment gangrenous ulcers from pressure are of very rare occurrence in Rosenbach's wards.—*Deut Med., Wochenschr.*, 6, 1889.

**Morbus Basedowii cured by Pregnancy.**—A servant girl had chorea for ten years, during the last four of which she complained of palpitation, precordial anguish, pains radiating from the region of the heart to the left shoulder, slight paresis of the extremities, and increase in the size of the thyroid gland. All these symptoms increased steadily, so that in October, 1887, she had paresis of the lower extremities, large goitre, palpitation of the heart, nystagmus, great exophthalmos, etc.; in February, 1888, great improvement in all the symptoms was noted, at which time she was pregnant with an illegitimate child. Mentally the pregnancy could not have exerted other than a depressing influence on the girl.—*Cent. fur Gynec.*

**What are the thoughts of the Dying?**—In the Societe de Biologie Fere affirmed that a dying person in his last moments thinks of the chief events of his life. Persons resuscitated from drowning, epileptics with grave attacks, persons dying and already unconscious, but momentarily brought back to consciousness by ether injections to utter their last thoughts, all acknowledge that their last thoughts revert to momentous events of their life. Such an ether injection revives once more the normal disposition of cerebral activity, already nearly extinguished, and it might be possible at this moment to learn of certain events of the past life. Brown-Sequard mentions the remarkrble fact that persons who, in consequence of grave cerebral affections have been paralyzed for years, get back at once when dying their sensibility, mobility and intelligence. All such facts clearly show that at the moment of dissolution important changes take place, reacting upon the composition of the blood and the functions of the organs.—*Wien Med. Zetung.*

**The Morphine Habit.**—Erlenmeyer says that children born of women addicted to the morphine habit are practically morphine eaters from birth. During the first few days of life, unless morphine is given to them, they are very apt to suffer collapse; and this condition may end in death, the child being too weak to withstand the violent symptoms, which are similar to those which follow the sudden withdrawal of the drug in adult opium-eaters.—*Science.*

**The Etiology of Exophthalmic Goitre.**—At last we have something definite and noteworthy in reference to this vexed question. Dr. White (*Brit. Med. Jour.*, March 30th), in a case, found numerous hemorrhages close under the floor of the fourth ventricle, near the nucleus of the sixth, and extending outward to the inner part of the restiform bodies. This is, to be sure, but a single case, and yet, as occurring here, these changes are sufficient and in a situation to account for the threefold nature of the symptoms. Though the hemorrhages were recent and the disease of long duration, Dr. White thinks the microscopic nature of the preceding affections of the fourth ventricle, rightly accounts for the exophthalmic goitre, the hemorrhages being a late result of the weakened and diseased tissues at that place. —*Polyclinic.*

**Mumps.**—Dauchez noticed that in mumps contagion took place, in three cases, after an interval of fifteen days, once seventeen days, and once nineteen days, after apparent infection. Dr. Dauchez concludes that the incubation period is fifteen days on an average, that infection takes place between the first and third day of the fever, and that isolation, to be effective, must not be less than seven days. That where isolation is not practicable the best course is to prescribe antiseptic mouth washes.—*Ib.*

**A New Source of Morphine.**—We noted some months ago that it was claimed that an alkaloid identical in every respect, had been isolated from the *Eschholzia Californica*, but expressed some doubts as to the truth of the claim. All doubt seems now to be removed by a report from Mm. Adrian and Bardet confirming the first reports, and pronouncing the alkaloid to be morphine. Beside a small quantity of this alkaloid, these gentlemen have also separated from eschholzia a more considerable quantity of another undescribed base, as well as indications of a glucoside, and this is consistent with the observation that an extract from the plant produces in cold-blooded animals a physiological effect not brought about by morphine even in large doses (*Bulletin de Therapeucique*). A number of physiological experiments upon animals with an aqueous and an alcoholic extract of the plant, together with the results of the use of the extracts on patients in the Cochin Hospital, are reported upon by Dr. Ter-Zakariant. He is of the opinion that the plant yields a very valuable soporific preparation, which is useful in certain cases as an anodyne, while it is free from the inconveniences attending the administration of morphine.—*Ib.*

**Ural—A New Hypnotic.**—Dr. Gustavo Poppi describes a product under the name of ural, formed by the combination of chloral hydrate with urethan, and recommends it as an excelllent hypnotic which in ordinary doses produces a physiologically quiet sleep more rapidly and of longer duration than any of the other known hypnotics. He claims that its use produces no disagreeable accidents and in therapeutic doses no disturbance of blood pressure; if, however, poisonous doses are given to dogs under these circumstances reduction of blood-pressure is the principal mode through which its toxic action is expressed. No discomfort or distress is produced on awakening from the sleep produced by ural, and Dr. Poppi states that its use may be persisted in for several days without inconvenience. He claims that he has given it in different cases of heart-disease, hysteria, and other nervous affections, in all cases with the best results, even when other hypnotics had proved ineffective.—*Wiener Medizinische Wochenschrift.*

**Danger of Giving Chloroform by Gas-light.**—Ball (*The praetioner*), adds to the cases already reported a number of others showing the disagreeble and even dangerous symptoms that may arise, both to the patient and the bystanders, in using chloroform by gas-light. A strong irritant is produced under such circumstances, which has been found to be carbon oxychloride, or phosgene gas. The symptoms are paroxysms of coughing, a sense of choking and irritation in the throat, giddy feeling, and, after a time, a strangling and burning sensation over the larynx.—*Times and Register.*

**What Dressing shall lie next the Wound?**—Vaseline or oil spread upon any textile fabric represents the worse type of dressing, because the unguent mingles with the discharges and retards organization; because the textile fabric entangles new epithelium cells and connective-tissue cells, and because there is nothing in the dressing to prevent fermentation and wound infection. Lint and cotton are even worse than textile fabrics.

The cerates spread upon textile fabrics are one point better, in that new epithelium and connective tissue cells are not entangled in the mesh.

Balsams spread upon textile fabrics, or upon lint or cotton, are better than vaseline or cerate, because they may limit fermentation.

There are only two perfect types of dressing. The iodoform covering for small wounds represents one of these. Iodoform forms a thin, firm coagulum with lymph, and this is not easily attacked by micro-organisms. Beneath this coagulum the processes of repair go on smoothly in small wounds, even when a certain number of microbes are at work, because the iodoform neutralizes the ptomaines.

The other perfect dressing is the one suitable for larger wounds, and it possesses the following properties: First, smoothness, and impenetrability to new epithelium and connective-tissue cells (Lister's protective oiled silk). Second, a bulky mass which is highly absorptive, to draw serum away from the wound, and to make it too dry for microbe food; and this dressing is charged with antiseptics to destroy micro-organisms (bichloride cotton or gauze).—*Ib.*

**Astringent Washes** prevent the formation of bed-sores. Dr. Walker (*Med. Times and Reg.*) uses a one per cent. solution of chloral hydrate, with very beneficial results, in the treatment of bed-sores. The parts were thoroughly syringed, washed, and dressed in chloral.

Special care should be exercised by the physician to *prevent* the formation of bed-sores, by daily examination and prompt resort to astringent antiseptic dressings. Many a patient who has passed safely the crisis of a disease, has died from the fearful sufferings following upon the formation of bed-sores.—*Pop. Science News.*

Several years ago Dr. C. W. Prentice, of Washington, D. C., published a case in which he thought the use of jaborandi caused white hair to resume its natural dark color. He now reports another case, the patient being an old lady, with Bright's disease.—*Ib.*

**Sugar in the Urine.**—Dr. R. E. Haughton, of Indianapolis says: Sugar finds its way in the urine. Where does the sugar arise? Physiologists tell us that the sugar is manufactured by the liver, and from thence it passes into the blood. It is eliminated by the kidneys. I have used Lithiated Hydrangea, and I know it will control the formation of sugar. Whether it prevents the conversion of starch into sugar, or exercises its power in some other way, I am not prepared to say. —*Med. Brief.*

**Abortion.**—I well remember how many anxious hours have been spent, in former years, over cases of retention of afterbirth in abortion, how I have smudged my hands and hurt the woman, trying to get my fingers into a closed uterus, fearing some dreadful calamity if the woman was not speedily delivered. Now-a-days, I order large vaginal injections of hot water, with some listerine or carbolic acid, repeated three or four times a day, according to the urgency of the symptoms, and *wait.*

The hot water controls the hemorrhage perfectly, and after awhile everything comes away. I have twice waited till the third day, but saw no bad results from this delay.—Dr. Andrews in *Med. Brief.*

**Morphine as an Anti-Emetic.**—A writer in *Med. Brief* says that "when a child's stomach rejects everything, even the breakfast milk as if it were ipecac, try a little morphine dissolved in aromatic sulphuric acid, every fifteen minutes, and you will be agreeably surprised at the result."

**Action of Antipyrin on the Teeth.**—Among the inconveniences ascribed to the use of antipyrine, Dr. Galippe has brought to notice that in several cases in which the drug was administered internally the teeth were blackened by it. Dr. Galippe, who devotes himself to dentistry, could oger no scientific explanation of the manner in which antipyrine acts on the teeth; he, however, found that the teeth blacken more readily when they have lost their enamel. But this inconvenience is only transitory, and may be removed by simply rubbbing the teeth with oxygenated water.—*London Lancet.*

Dr. Charles F. Mason, Acting Assistant Surgeon, U. S. A., writes: "In a recent trip from New York to Norfolk by sea, I had an opportunity of observing, both in myself and others, the remarkable curative power of cocaine in sea-sckness. The cases treated, four in number, were all adults, two being physicians. The symptoms in all were extreme nausea and great depression; no vomiting. Cocaine hydrochlorate, in doses of twelve minims of a four per cent. aqueous solution afforded almost complete relief in from fifteen to thirty minutes, so that the three patients who were below were enabled to dress and come on deck. After a second half-grain dose (two hours' interval) the relief was complete and permanent.—*N. Y. Med. Times.*

**The Period of Incubation in Eruptive Fevers.**—

*Measles.*—Incubations, ten to fourteen a day. Rash appears fourth day of the fever; fades on the seventh day of the fever. Duration of the disease, six to ten days.

*Rotheln.*—Incubation six to ten days. Rash appears twelve to thirty-six hours after fever; fades twenty-four to forty-eight hours. Duration, three or four days.

*Scarlet Fever.*—Incubation, one to six days; occasionally as far as twenty-one days. Eruption on the sęcond day of fever; fades on fifth day of fever. Duration, eight to ten days.

*Small-Pox.*—Incubation, ten to fourteen days. Eruption on third day of fever. Scabs form on the ninth day; they fall off about the fourteenth day. Duration of the disease, fourteen to twenty-one days.

*Typhus Fever.*—Period of incubation, one to twelve days. Eruption appears fourth or seventh day. Duration, fourteen to twenty-one days.

*Typhoid Fever.*—Incubation, ten to fourteen days. Eruption, seventh to fourteenth day. Duration of the disease, twenty to thirty days.

*Chicken-Pox.*—Incubation, four days. Eruption, second day of fever, Duration, six to seven days.

*Erysipelas.*—Incubation, three to seven days. Eruption second or third day after fever; fades from twenty-four to forty-eight hours in simple cases; in bad cases the duration is indefinite.—*American Medical Journal.*

**Neutralizing Cobra Poison.**—About three months ago a number of snakes, principally cobras, were sent to Dr. Vincent Richards for experiment. Dr. Richards took out one of the cobras with the object of getting some of its poison in a watch glass, which he had ready for the purpose. He held the snake in his right hand, and was moving his left hand in front of it when the snake bit him severely on the forefinger of the left hand. Dr. Richards, fortunately for himself, preserved his presence of mind; he killed the snake, and with a knife laid the finger open to the bone above the wound and applied permanganate of potash; he then applied a ligature to the finger and another to the forearm, and drove off for medical advice. Dr. Macleod and Sir Benjamin Simpson reopened the wound and thoroughly cauterized it with nitric acid. Ordinary dressings were then put on. The *Indian Daily News* reports that no serious results followed the bite, and that Richards is now practically well.—*Ib.*

**A Chinese Anæsthetic.**—A curious anæsthetic used by the Chinese has recently been made known by Dr. U. Lambuth in his third annual report of the Soochow Hospital. It is obtained by placing a frog in a jar of flour and irritating it by prodding it. Under these circumstances it exudes a liquid, which forms a paste with a portion of the flour. This paste, dissolved in water, was found to possess well-marked anæsthetic properties. After the finger had been immersed in the liquid for a few minutes it could be pricked with a needle without being felt, and numbness of the lips and tongue was produced by applying the liquid to them.—*Boston Journal.*

**Myrtol.**—Myrtol is obtained from the distillation of the myrtle; it is a liquid possessing the characteristic perfume of the plant. It is of less density than water, evaporates at ordinary temperatures, stains paper, but the stains disappear entirely. It has a slightly acrid taste, soon followed by a sensation of freshness. It is said to be an excellent disinfectant and an energetic antiseptic, to stimulate the digestive functions, and to increase the appetite. In moderate doses, myrtol acts as a sedative to the nervous system. It is eliminated by the urinary and respiratory passages. In order to obtain the best results, it should be employed with a view to combating subacute or chronic bronchitis, or it may be given at the termination of an acute attack of bronchitis when the fever has subsided. Another indication for its employment is an abundant opaque mucu-purulent secretion. In these cases, the secretion is diminisned and rendered less purulent. M. Linaris has employed Myrtol in chronic fetid bronchitis, catarrhal bronchitis, catarrhal asthma with paroxysmal attacks and palpitations, capillary bronchitis and dilated bronchi. M. Linaris recommends the use of globules of myrtol each containing 15 centigrammes. The average daily dose should be 6 globules, taken after meals, 2 in the morning, 2 at noon, and 2 at eventide.—*Schu and Fink Notes.*

**Ice in the Sick Room.**—A saucerful of shaved ice may be preserved for 24 hours with the thermometer in the room at 90 F., if the following precautions are observed: Put the saucer containing the ice in a soup plate and cover it with another. Place the soup plate thus arranged on a good, heavy pillow, and cover it with another pillow, pressing the pillows so that the plates are completely embedded in them. An old jack-plane set deep is a most excellent thing with which to shave the ice. It should be turned bottom upward, and the ice shoved backward and foward over the cutter.—*N. Y. Med. Times.*

**Salt in milk for Children.**—Dr. A. Jacobi (*Arch. of Pedriatics*) says that the addition of sodium chloride prevents the solid coagulation of milk by either rennet or gastric juice. The cow's milk ought never to be given without table salt, and the latter ought to be added to woman's milk when it behaves like cow's milk in regard to solid curdling and consequent indigestibility. Habitual constipation of children is influenced beneficially, since not only is the food made more digestible, but the alimentary secretions, both serous and glandular, are made more effective by its presence.—*Ec. Med. Jour.*

**Salicylic Acid—the Indications for it.**—I imagine the majority of physicians would say, "There can be no difficulty in knowing when to give salicylic acid—give it in rheumatism." This has not been my experience in its use, and I commenced with its first discovery. I have seen some marvelous cures, I have seen complete failures, and I have seen unpleasant results. I have endeavored to make a study of it, and to determine when it would prove a remedy, and two or three times, when I thought I had the problem solved, I found I had failed.

I believe that salicylic acid from wintergreen is decidedly preferable to that from carbolic acid as an anti-rheumatic, a remedy for neuralgia, or an antipyretic. One thing is very certain: it is less irritant to the stomach. There are cases in which salicylic acid is best given in pills, two grains at a dose—it is used as an acid. This gave the marked cures of the first two years of its use. Then it acted best as a salicylate of soda or potash, in solution. I prefer the salicylate of potash, and make the dose contain about two grains of the acid.

I have two rules that guide me in prescribing it. If the breath is markedly fetid give salicylic acid or a salicylate. If the tongue is purplish in color, large and coated in the centre, give salicylate of potash.

With so many physicians using the remedy it should not be difficult at this time to determine the exact case where the remedy will prove curative. It may be that I have not looked in the right direction to find the indications for it, and others may have been more successful. If so, I should like to hear from them.—*Ibid.*

**The American Medical Association.**—The meeting at Newport differed widely from its immediate predecessors. There was a small attendance; not over seven hundred and fifty registering at the outside. This was due to several causes combining. The Old Colony Railroad refused to make special rates, and the other roads consequently withdrew their offers to do so. Then, at the last moment, the Old Colony announced the reduction, when it was too late to take advantage of it, or to get the other roads to do likewise. The road ought to be boycotted by every friend of the Association.

The second element determining the small attendance was the absence of anything like a fight. We are very scientific, especially in the East, but we still take interest in a struggle for superiority.

Finally, the town of Newport presents no attractions to the physician, such as are to be found in the great Medical centers—colleges, hospitals, clinics, and the great teachers of the day. In all respects our predictions in regard to Newport were fulfilled. The one hotel was crowded to repletion; so that many were unable to obtain even one of the antideluvian beds which almost completely filled up the cells in which we were to spend the nights. The cooking department broke down; so that guests were compelled to wait in front of the dining-room door for over an hour after the Sections opened their meetings before they could be served. For this, Newport could hardly be blamed, as she never made any pretense of being a city of hotels. It is to be hoped that things will be better in Nashville next year.

The address of President Dawson dealt with medical education, medical literature, and the achievements of modern surgery. He discussed at some length the question of legislation upon medical matters, and opposed the creation of Examining Boards composed in part of irregulars.

The address in Surgery was a feature of the meeting. Dr. Conner had certainly an abundance of material in the surgical work of the present generation, and he used it judiciously. He protested against the extravagant hopes which see in failure only an evidence of neglect on the surgeon's part, and recommended a collective investigation upon the subject of carcinoma.

The address in medicine was a disappointment. Those who had heard the brilliant clinical teacher of the University of Pennsylvania, looked for something in this Address which would in itself repay them for the journey and the inconveniences of the sojourn at Newport. Instead of this, they heard a stale biography of Rush. Dr. Rush was a very worthy gentleman, and we sincerely hope Dr. Gihon will succeed in raising a monument to his memory; but that is no reason why he should be foisted upon an audience which had collected from all parts of the country to hear Dr. William Pepper's views upon modern medicine.

In the selection of Dr. E. M. Moore, of Rochester, as the next President, the Association honored itself and testified its appreciation of his work, his services to the profession, and his devotion to the welfare of the Association. We have been credibly informed that the candidate put foward in opposition to Dr. Moore failed to get a single vote outside of his own State; but whether this arose from the popularity of the one or the dislike of the other we are unable to say. The direction of the Journal is left in the same capable hands as during the past few months; the trustees being strengthened by the addition of Drs. Hooper, Garcelon, Love and Dawson. If the only question in this connection were the efficient management of the publication, there would be little to object to in this arrangement. But as long as there is no ostensible head to the Journal there will be intriguing and wire-pulling by persons who desire the post of managing editor. Some of these parties will do all in their power to embarrass the present management in order that their own selfish interests may be subserved. Factions will endeavor to thrust into the seat men who have been their tools in times past. These annoyances would have been prevented by the selection of a strong man to fill the chair. One cannot but admire the effrontery with which some of these men urge their alleged claims. Editors who have been persistent enemies to the Association, or who have been the most egregious failures in every sense, consider themselves fit to fill N. S. Davis' shoes!

The social features were not so elaborate as on former occasions. By far the most enjoyable was the excursion of the Medical Editors' Association, which was in all respects a success, and will long be remembered by the participants. But one unpleasant incident marred the harmony of the occasion. By some mischance, the last toast was assigned to a person who represented a pharmaceutical journal's advertising interests; and this man had so little conception of the amenities of civilized life that he embraced this opportunity, at a social gathering, to make a bitter attack upon an enterprise recently launched by one of the guests present, for which he freely predicted bankruptcy. The address was heard with surprise, which would have assumed the form of active indignation were it not that the insinuations were couched in such obscure terms that few understood what the speaker was driving at. It is to be hoped that in future pharmacy will be somewhat more worthily represented. As the *Druggists' Circular* is in no way effected by the success or failure of the scheme referred to, it is evident that its advertising scheme was simply put forward by some parties whose interests are more directly influenced; and who chose as their representative a man who was the only one connected with a pharmaceutical journal who could be induced to do their dirty work. That this was done with the deliberate intention of doing an injury to the project in question, is shown by the fact that several slips containing the remarks in question were printed and mailed to the medical journals on the Saturday following. It is evident that somebody is pretty badly scared at the favor with which the consolidation journal has been received.

The clam bake tendered to the Association by the Rhode Island Medical Society was a very elaborate affair, and was enjoyed by the guests.—*Ex.*

**A Combination of Narcotics and Exhilarants.**—Dr. J. B. Johnson in *Southern Clinic* says: The following combination of narcotics and exhilirants, I have found to act more satisfactorily than when prescribed separately, in that large class of nervous, neuralgic and rheumatic cases, which are not in themselves dangerous to life, but are a source of great complaint from the sufferers from them, and for which physicians are so often called upon to prescribe:

℞ Tinct. belladonna leaves,
Tinct. aconite root,
Tinct. cannabis indica,
Tinct. colchicum seed,
Tinct. cimicifuga,
Tinct. gelsemium ää ʒj.
Alcohol ʒiij.

M. S. Shake well. Dose, ten to thirty drops every three or four hours.

I have often been much pleased to find how rapidly many of my patients have been relieved of their chronic rheumatic, neuralgic and nervous pains by this prescription. Should constipation exist, it may be easily overcome by eight to ten drop doses of fluid ext. cascara sagrada, given in any agreeable vehicle like fluid ext. liquorice root.

**The Prevention of Conception.**—At the Third General Meeting of Russian Medical Men at St. Petersburg, Dr. Petr N. Seilder read an interesting paper on the truly burning subject of conception. While condemning an indiscriminate employment of any preventive means, the author believes that the practitioner is fully justified in interfering with conception in the following three categories of cases: 1. In women suffering with a more or less advanced pulmonary phthisis. 2. In women with organic cardiac disease. 3. In women suffering from profound anæmia, or failure of the general systemic nutrition with a hereditary tendency to pulmonary tuberculosis.

During a discussion following Dr. Seilder's communication Dr. Nil I. Voblyi suggested that prevention is indicated, further, in such women as have once or oftener passed through an extra-uterine pregnancy.—*Med. & Surg. Reporter.*

**The Use of Rhus Aromaticus in Incontinence of Urine.**—According to a recent number of the Union Medicale, quoting from the Archives Medicales Belges, Dr. Burdenich, of Gand, has tried *Rhus aromaticus* in 33 cases. He obtained an excellent result in 11, a satisfactory result in 10, and a result of little importance in the remaining 12 cases. The good effects of the medicine were not felt until the fifth or sixth day, and sometimes not even until the end of the third or fourth week. Dr. Burdenich considers rhus aromaticus to be a powerful tonic analagous to nux vomica. Given in doses of about 38 grains a day, it rendered urination easy and continuous in the case of a man 75 years old, who had partial paralysis of the bladder without contraction of the urethra or hypertrophy of the prostate. Dr. Numa, who thinks that the drug excites muscular action in the bladder, the uterus, and the lower part of the intestinal canal, gives 10 drops of the tincture to children from two to six years old, and 15 drops twice a day to older children. He thinks that the tonic effects do not persist. Dr. Hamon has succeeded in preventing children from urinating in bed by giving them a dose of from 20 to 60 drops of the tincture at bedtime.—*Med. Age.*

**Rectal Troubles.**—One word respecting the value of anodyne applications. The distressing pain which attends the evacuation of the bowels in some diseases, and continues for hours after, often demands local palliative treatment. Many cases of pain and spasm of the orifice are greatly relieved by the topical use of cocaine; but, after many trials, I found a combination of morphine and cocaine more generally useful. The application of this compound sedative often acts like a charm, and quickly gives relief. The suffering caused by fissure of the anus and ulceration within the spinchter is rendered bearable by this remedy, and in recent cases I have sometimes found its use, combined with digital dilation, rapidly followed by permanent cure, without any other surgical interference. It is invaluable in the treatment of stricture of the rectum, and it should be introduced into the bowel ten minutes before the use of the dilator. In chronic ulceration and malignant disease it can be freely employed as a palliative remedy and, in fact, in all cases in which pain and hyperæsthesia are prominent symptoms.—*Provincial Med. Jour.*, Halifax, Eng.

**A New Use for Ether During Anæsthesia.**—Very frequently during the early stages of the administration of anæsthetic the patients "forget to breathe," even before the ability to perceive peripheral irritation is lost. Even later in anæsthesia, when breathing suddenly ceases we are accustomed to use cold water externally and to slap the patient with wet towels.

Such measures are generally called for hurriedly, and it is not all uncommon for an exasperating delay to occur before the water arrives. The ether is always at hand, however, and I have found that in a large number of instances, both in man and the lower animals, the free use of ether poured upon the belly causes so great a shock by the cold produced by its evaporation as to cause a very deep inspiration, which is often followed by the normal respiratory movements. This is, of course, a simple procedure, and one which has probably been used by others, but I have never seen it so employed.—H. A. Hare, M. D., Demonstrator of Therapeutics, University of Pennsylvania, *University Magazine.*

